# The effectiveness of a semi-tailored facilitator-based intervention to optimise chronic care management in general practice: a stepped-wedge randomised controlled trial

**DOI:** 10.1186/1471-2296-15-65

**Published:** 2014-04-09

**Authors:** Tina Drud Due, Thorkil Thorsen, Marius Brostrøm Kousgaard, Volkert Dirk Siersma, Frans Boch Waldorff

**Affiliations:** 1 The Research Unit for General Practice and Section of General Practice, Department of Public Health, University of Copenhagen, Copenhagen, Denmark

**Keywords:** Disease management programmes, Facilitation, Implementation, RCT, Outreach visits, General practice, Diabetes, COPD

## Abstract

**Background:**

The Danish health care sector is reorganising based on disease management programmes designed to secure integrated and high quality chronic care across hospitals, general practitioners and municipalities. The disease management programmes assign a central role to general practice; and in the Capital Region of Denmark a facilitator-based intervention was undertaken to support the implementation of the programmes in general practice. The purpose of the study was to assess the effectiveness of this semi-tailored facilitator-based intervention.

**Method:**

The study was a stepped-wedge, randomised, controlled trial among general practices in the Capital Region of Denmark. The intervention group was offered three one-hour visits by a facilitator. The intervention was semi-tailored to the perceived needs as defined by each general practice, and the practices could choose from a list of possible topics. The control group was a delayed intervention group. The primary outcome was change in the number of annual chronic disease check-ups. Secondary outcomes were: changes in the number of annual check-ups for type 2 diabetes (DM2) and chronic obstructive pulmonary disease (COPD); changes in the number of spirometry tests, changes in the use of ICPC diagnosis coding and patient stratification; sign-up for a software program for patient overview; and reduction in number of practices with few annual chronic disease check-ups.

**Results:**

We randomised 189 general practices: 96 practices were allocated to the intervention group and 93 to the delayed intervention group. For the primary outcome, 94 and 89 practices were analysed. Almost every outcome improved from baseline to follow-up in both allocation groups. At follow-up there was no difference between allocation groups for the primary outcome (p = 0.1639). However, some secondary outcomes favoured the intervention: a higher reported use of ICPC diagnosis coding for DM2 and COPD (p = 0.0050, p = 0.0243 respectively), stratification for COPD (p = 0.0185) and a faster initial sign-up rate for the software program.

**Conclusion:**

The mixed results from this study indicate that a semi-tailored facilitator-based intervention of relatively low intensity is unlikely to add substantially to the implementation of disease management programmes for DM2 and COPD in a context marked by important concurrent initiatives (including financial incentives and mandatory registry participation) aimed at moving all practices towards changes in chronic care.

**Trial registration:**

ClinicalTrials.gov: NCT01297075

## Background

About a third of the Danish population has at least one chronic disease and 70–80% of the resources in the Danish health care sector are used on these patients [1]. Disease management programmes (DMPs) based on the Chronic Care Model [2] have been developed in all regions of Denmark [3]. The DMPs outline a systematic, proactive approach to chronic care including a division of tasks between general practitioners, hospitals and municipalities. The programmes stress the need for population-based patient registration; annual chronic disease check-ups; and stratification of patients into three levels according to risk of complications, complexity, and state of the disease [4,5].

The implementation of DMPs is complex and may be aided by facilitator-based interventions consisting of outreach visits in general practice by an external person providing information and support. Facilitator-based interventions have been used to support development and implementation of guidelines in general practice since the 1980s in England [6,7] and have since been used in numerous interventions internationally and in Denmark [6-35]. Several studies have shown that facilitator-based interventions have contributed to changes in general practice [11,19,23,26,32,36], while others have found no effect [20,22,28]. Although previous studies are very heterogeneous in terms of content and intensity of the interventions and the role of the facilitator, a new systematic review and meta-analysis of practice facilitation concludes that facilitation has a moderately robust effect on the adoption of guidelines [11]. A recent Cochrane review of educational outreach visits, a concept closely related to facilitation, concludes that the most consistent effects concern the prescribing of medicine, while the effects of facilitation are less clear and more varied in other areas such as prevention [25]. There are only few randomised controlled trials of interventions using practice facilitation as an implementation strategy to enhance the implementation of the chronic disease management [37,38]. Thus, the aim of this study was to examine the effectiveness of a semi-tailored facilitator-based intervention developed by the Capital Region of Denmark to support the implementation in general practice of DMPs for chronic obstructive pulmonary disease (COPD) and type 2 diabetes (DM2).

## Method

### Setting

The Danish health care system is primarily tax financed and offers residents free-of-charge access to general practice (GP) and public hospital services. Almost all residents (98%) are registered with a GP, who serves as the primary care provider and a gatekeeper for patients’ referral to specialists and hospitals. The study population was all 762 general practices in the Capital Region of Denmark on 1 December 2010. We consecutively included practices that signed up for facilitation visits and completed a baseline questionnaire from January 2011 until we reached the 189 practices needed for the study. We excluded practices in which a facilitator worked and practices that had participated in a pilot study.

### Study design

The study is a stepped-wedge, randomised, controlled trial (RCT) where the intervention is rolled out to groups sequentially and data are collected at baseline and before new groups receive the intervention. Groups that have not yet received the intervention thereby function as control groups [39]. The stepped-wedge design has been used in several studies in the health care sector and is perceived as relevant either for ethical or practical/logistical reasons [40].

The intervention was developed and implemented by the Region. The outcome measures were chosen in collaboration between the Region and the researchers. The design and execution of the RCT and the data analysis were performed independently by the researchers.

The practices were randomly allocated to facilitator visits in 2011 (intervention) or to facilitator visits in 2012 (ratio 1:1).

### Ethics

The Danish Data Protection Agency and the Health Research Ethics Committee were informed about the study and found it unnecessary to report (j.nr. 2011-41-5958 and H-C-FSP-2011-04). The study is reported in ClinicalTrials.gov (NCT01297075). The Committee of Multi Practice Studies in General Practice in Denmark approved the study and recommended GPs’ to participate. (MPU 7–2011).

### The intervention

The aim of the intervention was to support the implementation in general practice of the disease management programmes for COPD and DM2. The intervention focused on the GPs’ role as coordinator of care, patient stratification, a proactive approach, and a systematic organisation of the workflow and division of tasks in general practice concerning chronic disease check-ups. The intervention was semi-tailored to the perceived needs as defined by each general practice.

The intervention consisted of two phases: 1) the facilitator education and development of a toolbox and 2) the facilitator visits.

#### Facilitators

A total of 14 general practitioners (GPs) were recruited as facilitators as well as one organisational consultant. One of the 14 GPs had an additional education in organisational development. The facilitators were remunerated for participating in the development of the intervention and subsequently according to their number of visits.

#### Facilitator education and development of a toolbox

The training of the facilitators was multifaceted and consisted of an educational programme, workgroups, a pilot phase and on-going network meetings.

The educational programme consisted of about 40 hours of meetings (a weekend seminar and 10 three-hour meetings) and was composed of two major activities: a professional update in relation to the DMPs and related tools; and education on the act of facilitation including coaching, communication skills, meeting management and development processes.

Concurrently, the facilitators met in workgroups and developed various tools for use during the visits (e.g. PowerPoint slides introducing the different topics, descriptions of methods applicable to the facilitator, relevant brochures for hand-outs and practice management development tools). Each facilitator used 10 hours to develop the toolbox.

In a pilot phase, the facilitators practiced their skills and tools in 11 selected practices.

During the project period, three-hourly network meetings were held quarterly. The meetings aimed at further education, adjustments of the tools, and discussions among the facilitators concerning their role and experiences at the visits.

#### The facilitator visits

All GPs in the Region were informed about the intervention via postal letters, news mails, professional meetings and advertisements on the Region’s web pages. Each participating practice was offered up to three facilitator visits of each one hour. The visits were free of charge and the practice was compensated for lost income (approx. €200 per doctor in the practice per visit).

##### Before the visits

Before the allocation, the practices completed an online baseline questionnaire containing items about practice characteristics, annual check-ups for DM2 and COPD, diagnosis coding, patient stratification, and use of a software program for patient overview and quality data (Sentinel Data Capture). Close to the first visit, each practice completed a second online questionnaire that focused on their knowledge of the DMPs, division of tasks in the practice, collaboration with the municipalities, and suggestions for topics for the visits. A report containing the responses was generated and sent to the facilitator and the practice. The questionnaires provided the facilitators with an impression of the developmental state of the practice in relation to the DMPs, and this aided them in selecting potentially relevant elements from the toolbox. The responses could be brought into the discussions at the visits, but the questionnaires were not meant as an audit tool.

Prior to the first visit, the facilitator contacted the practice regarding the choice of topics to be discussed. The practice could choose one or more topics from a range of predefined topics within the scope of the DMPs for COPD and DM2:

• Workflow and internal division of tasks in relation to regular and annual chronic disease check-ups

• The software program for patient overview and quality data (Sentinel Data Capture) [41]

• Diagnosis coding

• Stratification of patients

• Leadership and organisation

• Collaboration with municipalities and hospitals

• The role of GPs as coordinators of care

##### The visits

It was the intention that all doctors and staff members in the practice participated during the visits.

At the first visit, the facilitators were to assist in defining goals for practice development and in choosing suitable means to achieve them. During the subsequent meetings, the facilitators were to encourage and support a process of change. They were to act as discussion partners and colleagues rather than experts. They could also demonstrate relevant instruments for achieving the goals and recommend courses or other consultants for more specialised assistance.

After each visit the facilitator provided the practice with a standardised visit report containing the topics discussed and the goals and tasks agreed upon at the visit. It was a central element of the intervention that the practice was to work with the chosen tasks in the time until the next visit.

### Outcome measures

Data were retrieved from the Danish National Health Service Register (DNHSR) [42], the Danish Quality Unit of General Practice [41] and from questionnaires. The DNHSR is used to manage the National Health Insurance covering the primary health care sector and, in particular, is used for settling accounts with providers.

**Figure 1:**
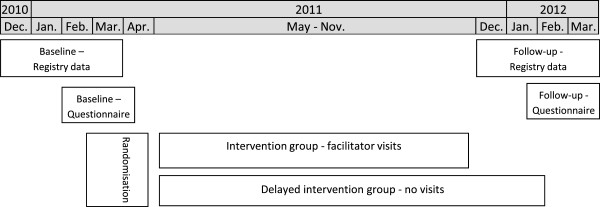
Timetable.

The questionnaires were tested for relevance and applicability in a pilot study with 11 practices. The baseline questionnaires were collected before randomisation, and registry data were collected for each practice in the three months up to randomisation. The follow-up registry data were collected a year later, in the three months equivalent to the baseline period. The follow-up questionnaires were sent out on 15 February 2012. Thus, the follow-up was 12 months and the intervention 9 months. The delayed intervention group did not receive the intervention until after the follow-up registry data and questionnaires had been collected (Figure 1).

#### The primary outcome

1. Change in the number of annual chronic disease check-ups per 100 patients affiliated with the practice (DNHSR).

The annual chronic disease check-up is a structured consultation dealing with the status and progression of the disease and the disease-specific risk factors. The check-up also includes a dialogue about the patient’s wellbeing, illness experience, lifestyle and medical treatment. Lastly, it covers patient stratification and an assessment of the need for adjusting treatment and/or treatment goals. Stratification is a grouping of patients based upon their care needs’. It consists of an assessment of the current severity and complexity of the disease (including risk of complications and comorbidity) and an assessment of the patient’s capacity for self-care. The stratification is used to divide the responsibility for treatment between the general practitioner, the hospital and the municipality.

The annual chronic disease check-up is a core element in all DMPs [3], and we hypothesised that the facilitator visits would increase the use of annual chronic disease check-ups. Therefore, this was chosen as proxy measure of the implementation of the systematic approach to chronic care as emphasised in the programmes. We were able to retrieve this information directly from the DNHSR, independently of GPs’ assessments.

#### The secondary outcomes

2. Reduction in the number of practices with few (less than 1%) annual chronic disease check-ups per 100 patients affiliated with the practice (DNHSR).

3. Change in the number of spirometry tests per 100 patients affiliated with the practice (DNHSR).

4. Change in the number of annual check-ups for DM2 and COPD (self-reported).

5. Sign-up to the Sentinel Data Capture (a software program that automatically collects patient data from the GPs’ electronic health record system) [41] (register based).

6. Changes in the use of ICPC diagnosis coding for DM2 and COPD (self-reported).

7. Changes in the use of stratification of patients with DM2 and COPD (self-reported).

We hypothesised that the intervention would decrease the number of practices with few annual chronic disease check-ups (outcome measure 2), and increase the uptake of several tools indicative of a more systematic approach to chronic care (outcome measure: 3, 4, 5, 6 and 7), because these tools are core elements of the DMPs and were among the optional topics for the visits.

### Sample size

The power calculation was based on data from the Danish National Health Service Register on the use of annual chronic disease check-ups during 1 January 2010–1 December 2010. Two parameters were calculated.

1. To increase the number of annual chronic disease check-ups from 1.25 to 2.0 consultations per 100 patients per quarter.

2. To reduce the number of practices with less than 1% annual chronic disease check-ups per 100 patients per quarter from 40% to 20%.

In accordance with the first parameter, there was a need for 128 practices at a power of 80% and a significance level of 5%. Using the second parameter, there was a need for 163 practices at the same power level. We estimated a dropout rate of 10% and therefore included 189 practices.

### Randomisation and allocation concealment

Eligible practices were stratified by practice type (solo or group practice) and geographical location by using SAS version 9.2. The allocation of practices was done by an external organisation (Danish College of General Practitioners) independently of the research group. Due to the nature of the intervention it was not possible to conceal the intervention for the practices and the facilitators.

### Statistical methods

Differences in the use of annual chronic disease check-ups and spirometry between allocation groups were assessed using t-tests. Differences between allocation groups regarding the rest of the outcome measures were assessed using chi-squared tests. All differences were assessed at baseline and at follow-up separately. The difference in sign-up rate to the Sentinel Data Capture over time was visualised in a Kaplan-Meier plot and analysed with a log-rank test. All statistical analyses were done using SAS version 9.2 (SAS Institute Inc., Cary, NC).

## Results

### Trial flow

**Figure 2:**
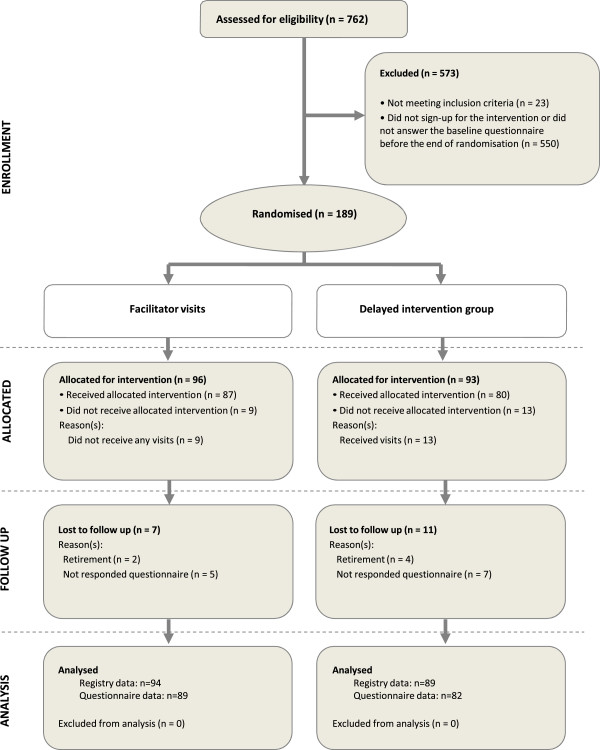
Trial flow.

Of the 762 practices in the Capital Region of Denmark, 189 were included in the RCT (Figure 2). There were no significant differences in practice characteristics or outcome measures between allocation groups at baseline (Table 1). Six practices dropped out of the study due to retirement and were excluded from the analysis and 12 practices did not answer the questionnaires and are represented only in the registry data. There were no differences in practice characteristics or baseline measures between the analysed practices and those lost to follow-up. In the delayed intervention group, 13 practices received visits during the intervention period, either because they were collaborating with practices in the intervention group or because they did not agree to delay their participation in the intervention.

**Table 1:** Practice characteristics and the distribution of outcomes at baseline

	**Intervention group (N = 96)**	**Delayed intervention group (N = 93)**	**P-value**
**Practice characteristics** (Regional registry data)			
Type of practice			0.7605
Solo	64%	67%	
Others	36%	33%	
Ratio of doctors to patients (median (IQR))	1513 (1245-1768)	1503 (1078-1730)	0.1979
Proportion of practices with nurses	77%	70%	0.3227
**Primary outcome measures** (The Danish National Health Service Register)			
Annual chronic disease check-ups per 100 patients affiliated with the practice N = 96/93 (median (IQR))	1.1 (0.4-2.4)	1.1 (0.3-2.3)	0.8372
**Secondary outcome measures**			
Number of practices with less than 1% annual chronic disease check-ups N = 96/93 (The Danish National Health Service Register)	47%	47%	1.00
Spirometry test per 100 patients affiliated with the practice N = 96/93 (The Danish National Health Service Register) (median (IQR))	0.66 (0.2-1.1)	0.5 (0.1-1.0)	0.7156
Annual check-ups for Diabetes N = 9 6/93 (Questionnaires)			0.8185
Yes – always	84%	87%	
Yes – sometimes	10%	8%	
No	5%	5%	
Annual check-ups for COPD N = 96/93 (Questionnaires)			0.6900
Yes – always	30%	30%	
Yes – sometimes	53%	48%	
No	17%	22%	
Sign-up for the Sentinel Data Capture N = 96/93 (Regional registry data (DAK-E))	25%	22%	0.6085
ICPC diagnosis coding – Diabetes N = 96/93 (Questionnaires)			0.2391
Yes – always	48%	40%	
Yes – sometimes	34%	32%	
No	17%	28%	
ICPC diagnosis coding – COPD N = 96/93 (Questionnaires)			0.1311
Yes – always	35%	26%	
Yes – sometimes	42%	39%	
No	23%	35%	
Stratification – Diabetes N = 96/93 (Questionnaires)			0.3283
Yes – always	14%	22%	
Yes – sometimes	36%	35%	
No	50%	43%	
Stratification – COPD N = 96/93 (Questionnaires)			0.9324
Yes – always	10%	11%	
Yes – sometimes	36%	33%	
No	53%	56%	

The N at each outcome measure shows the number of practices in the analysis for the intervention/delayed intervention group.

### Implementation of the intervention

Each practice could choose up to three one-hour visits. Seven practices divided the three hours into two visits; hence, the number of hours visited is used instead of number of visits. In the intervention group, 178 visits were undertaken during the intervention period. Of the 96 practices in the intervention group, 24% received all three hours of facilitation, 39% received two hours’, 29% received one hour and 9% had no visit. Some practices chose not to have more visits and others did not finish their visits during the intervention period but continued afterwards (46 of the 96 practices).

According to the visit reports made by the facilitators, several topics were usually discussed at the visits, but the number of topics typically decreased from the first to the third visit. The most common topic was the Sentinel Data Capture; other common topics were diagnosis coding, stratification, a website on municipal health services, and division of tasks.

### Effectiveness of the intervention

With a few exceptions, all outcome measures improved from baseline to follow-up in both allocation groups (Tables 1 and 2).

**Table 2:** The distribution of outcomes at follow-up

	**Intervention group (2011) (N = 94)**	**Delayed intervention group (2012) (N = 89)**	**P-value**
**Primary outcome measures**			
Annual chronic disease check-ups per 100 patients affiliated with the practice N = 94/89 (median (IQR))	1.9 (0.9-3.9)	1.7 (0.7-3.5)	0.2788
Change in annual chronic disease check-ups per 100 patients affiliated with the practice N = 94/89 (median (IQR))	0.5 (0.0-1.9)	0.5 (0.0-1.3)	0.1639
**Secondary outcome measures**			
Number of practices with less than 1% annual chronic disease check-ups N = 94/89	29%	33%	0.6314
Reduction in the number of practices with less than 1% annual chronic disease check-ups. N = 94/89	24%	18%	0.4403
Spirometry test per 100 patients affiliated with the practice N = 95/89 (median (IQR))	0.6 (0.2-1.2)	0.5 (0.1-0.8)	0.0835
Annual check-ups for Diabetes N = 89/82			0.2345
Yes – always	92%	88%	
Yes – sometimes	8%	9%	
No	0%	4%	
Annual check-ups for COPD N = 89/82			0.0787
Yes – always	53%	37%	
Yes – sometimes	39%	49%	
No	8%	15%	
Sign-up for the Sentinel Data Capture N = 94/89	71%	63%	0.2708
ICPC diagnosis coding – Diabetes N = 89/82			0.0050*
Yes – always	87%	67%	
Yes – sometimes	12%	27%	
No	1%	6%	
ICPC diagnosis coding – COPD N = 89/82			0.0243*
Yes – always	73%	57%	
Yes – sometimes	25%	32%	
No	2%	11%	
Stratification – Diabetes N = 89/82			0.0598
Yes – always	27%	13%	
Yes – sometimes	43%	44%	
No	30%	43%	
Stratification – COPD N = 89/82			0.0185*
Yes – always	24%	11%	
Yes – sometimes	45%	39%	
No	31%	50%	

The N at each outcome measure shows the number of practices in the analysis for the intervention/delayed intervention group.

*Indicate a significant difference between the allocations groups (P<0.05).

The use of annual chronic disease check-ups increased in both allocation groups (72% and 55% respectively), but there was no significant difference between the groups at follow-up (p = 0.2788) and no significant difference in the change from baseline to follow-up (p = 0.1639) (Table 2). There were no significant differences regarding the self-reported use of annual check-ups for DM2 and COPD (p = 0.2345; p = 0.0787 respectively), the reduction in the number of practices with few annual chronic disease check-ups (p = 0.4403), or in the use of spirometry (p = 0.0835).

The self-reported use of ICPC diagnosis coding was significantly in favour of the intervention group for both DM2 (p = 0.0050) and COPD (p = 0.0243). The difference in the proportion of practices that reported always using ICPC diagnosis coding at follow-up was 20 percentage points for DM2 and 16 percentage points for COPD.

The self-reported use of stratification was significantly different at follow-up for COPD (p = 0.0185) and there was a non-significant trend for DM2 (p = 0.0598) (Table 2). The difference between the allocation groups was, respectively, 13 percentage points for COPD and 14 percentage points for DM2 in the proportion of practices that reported always using stratification at follow-up.

**Figure 3:**
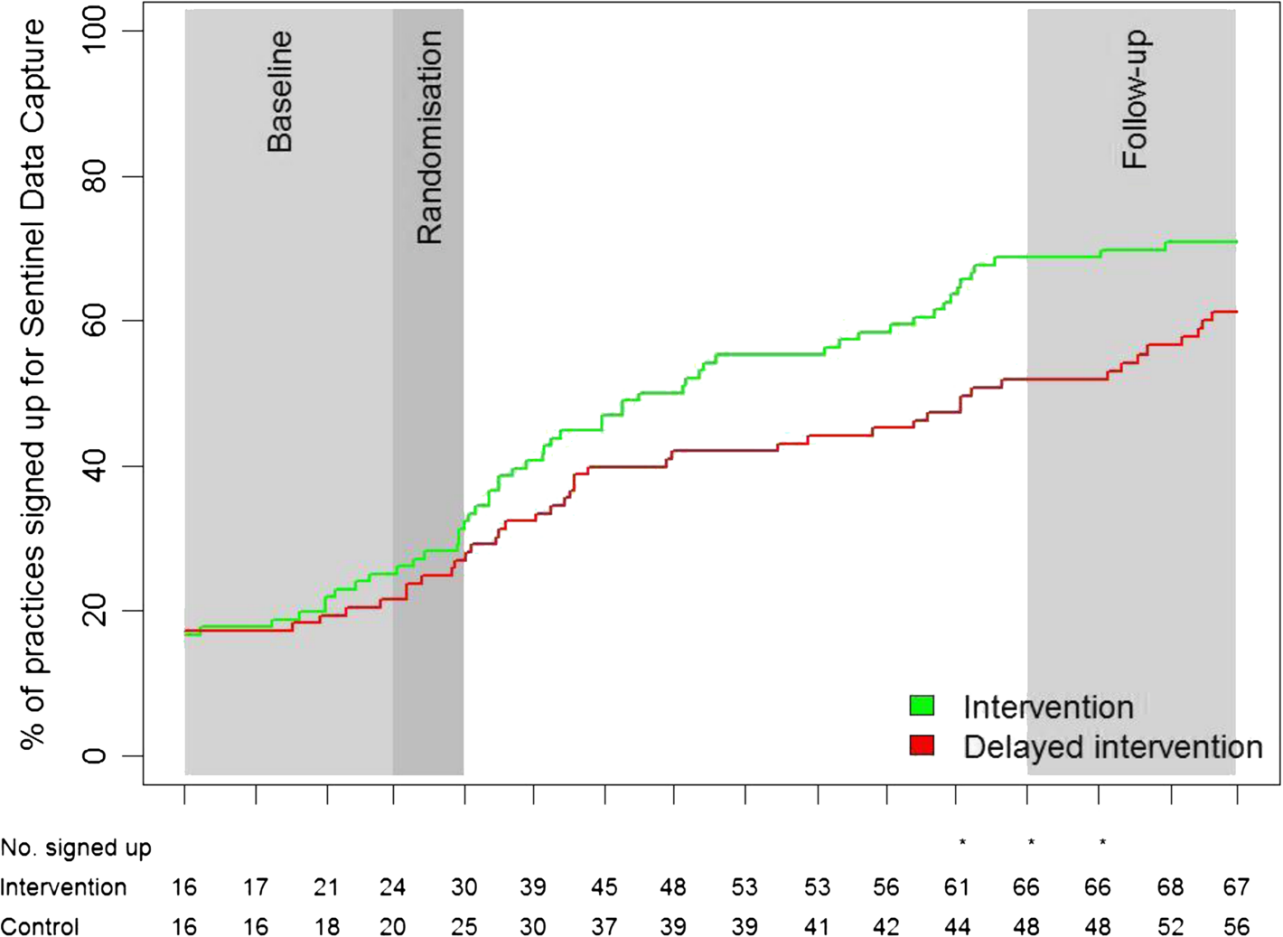
**Sign up for Sentinel Data Capture.** *Indicate a significant difference between the groups.

During the study period, the intervention group showed a faster sign-up rate to the Sentinel Data Capture; however, this did not result in a significant difference at the end of the study (Figure 3).

Because some practices in the intervention group received less than two hours of visits and some practices in the delayed intervention group received visits during the intervention period, we conducted a per protocol analysis comparing the practices in the intervention group that received a minimum of two hours’ visits (59 practices) with the practices in the delayed intervention group that received no visits (75 practices). In contrast to the prior results, there was no significant difference in the use of ICPC diagnosis coding for COPD in the per protocol analysis (p = 0.1104). However, the self-reported use of annual check-ups for COPD (p = 0.0232) and stratification for DM2 (p = 0.0026) became significantly different between the allocations groups.

## Discussion

In this large, stepped-wedge RCT of the effect of a semi-tailored facilitator intervention to support the implementation of disease management programmes for COPD and DM2 in general practice, we found an improvement in both allocation groups for nearly all outcome measures. However, when comparing the two allocation groups there was no additional effect of the intervention on the use of annual chronic disease check-ups, which was the primary outcome. Nevertheless, some of the self-reported secondary outcomes were in favour of the intervention, indicating a modest intervention effect.

In this study we used process-related rather than patient-related outcome measures. We did this because the objective of the study was to assess the effectiveness of the intervention as a tool to enhance the implementation of the disease management programmes.

The design of the intervention was characterised by a high degree of flexibility, for example, the latitude of the practices in selecting topics, the latitude of the facilitators in shaping their facilitation approach, and the absence of a detailed description for standardising the structure and content of the visits. This flexibility is likely to have generated wide variation across sites, thereby reducing the focus of the intervention and hence its impact on individual outcome measures. While the visit reports show that the practices chose to focus on similar topics, it is not possible to assess variations in how the topics were actually addressed or the time and level of detail spent on each topic. Conversely, the flexible design may have contributed to the positive impact of the intervention on some of the secondary outcomes by enhancing the motivation of the practices (by allowing them to choose the topics they found the most relevant), and by allowing the facilitators to tailor their approach to the particular situation of each practice.

That all outcome measures improved in both allocation groups from baseline to follow-up is probably influenced by chronic care and DMPs being highly profiled concepts in both the Capital Region of Denmark and the Danish health care system as a whole during the study period. The collective agreement between the Danish Regions and the GPs’ central organisation involves agreements and financial incentives for improving chronic care, and during the intervention period it became mandatory for all practices in Denmark to sign up for the Sentinel Data Capture before 31 March 2013. Further, several workshops and courses on Sentinel Data Capture, diagnosis coding, COPD and DM2 were provided in the Capital Region during the intervention period. These concomitant factors are highly likely to have influenced the study outcome. However, influences from the broader political context are inevitable when studying health services interventions in a real life setting, and hence this study underlines the importance of undertaking RCTs.

### Strengths of the study

Given that all outcome measures, with a few exceptions, improved from baseline to follow-up in both allocation groups, the study demonstrates the value of a randomised controlled trial design that exposes changes attributable to the intervention. If a simple before-after measurement had been applied, the effect would have been overestimated profoundly. In our study the stepped-wedge design was chosen because the facilitator-based intervention was a public intervention offered to all practices in the Capital Region of Denmark; as the Region assumed that the intervention would be beneficial, they did not consider a non-intervention group as an option.

We included a large number of practices and used a primary outcome that could be obtained from administrative registers independently of the participating practices. The dropout rate was relatively small and less than anticipated, which enhanced the power of the study.

### Limitations of the study

The practices included in this study were among the first ones to sign up for the intervention. Thus both allocation groups likely represent practices more interested in making changes, and this probably contributed to the improvements in both allocation groups.

The power calculations were based on an increase in annual chronic disease check-ups. The change in the intervention group equals the change used in the power calculations, but we did not take into account the large improvement in the delayed intervention group. This indicates that despite our study being large, it may be underpowered.

It is possible that the effect of the intervention was reduced by some practices in the intervention group not receiving the intended intervention and some in the delayed intervention group receiving visits during the intervention period. The per-protocol analysis revealed minor changes in the results regarding some of the secondary outcome measures. However, a per-protocol analysis does not adhere to the principles of an RCT since it imposes a risk of unknown bias. Moreover, when excluding practices not adhering to the protocol, the study no longer measures the effectiveness of the intervention in a real life setting but the effect on a more selected group of practices. Lastly, that the per protocol analysis did not change the results of the primary outcome suggests that even absolute fidelity to the allocated intervention would not have changed the results considerably.

To secure a high degree of reliability, we chose to use registry data for our primary outcome. The data in the DNHSR are gathered from the GPs’ electronic invoices to the Regional Health Administration. Due to this link with the reimbursement system, DNSHR data on the most common basic services are generally assumed to be complete, albeit no validity studies have been published [42]. Annual chronic disease check-ups are, however, not registered in the DNHSR according to the specific disease but only as a generic code. Thereby, our primary outcome refers to all annual chronic disease check-ups in the practice and not exclusively to DM2 and COPD. Nevertheless, the annual check-ups are an essential part of the DMPs, and we consider them a good indicator of whether the practice has implemented a more systematic approach to chronic care as described in the programmes. Further, we presume that an increase in the overall number of annual chronic disease check-ups also reflects an increase in annual check-ups for DM2 and COPD, since these are some of the major diseases for which the GPs provide annual chronic disease check-ups and are the two first diseases for which DMPs were developed. Additionally, data from the self-reported questionnaires concerning annual check-ups specifically for DM2 and COPD support the result of our primary outcome.

Several of the secondary outcome measures are self-reported and the significant results are among these. With the current data it is not possible to test the accuracy of the self-reported data information. Additionally, some parameters relevant in relation to the DMPs are not included in the study, for example, the content of the annual check-ups, and how the stratification is actually applied. Such data were not available in the study period.

### The generalizability of the study

The study is an effectiveness study of an intervention in a real life setting and this adds to the external validity and the possibility of generalising the result to other regions in Denmark. However, it is more difficult to generalize the results to health systems that are markedly different from the Danish setting in which GPs are private entrepreneurs primarily financed through Public Health Insurance and where the collective agreements with the health authorities play a significant role in shaping the services provided.

### Comparison with the literature

A recent systematic review [11] finds a positive effect of practice facilitation, whereas the recent Cochrane review of educational outreach visits finds small but consistent effects concerning the prescription of medicine but more mixed results for other areas such as prevention [25]. Individual studies also find an effect in some outcome measures but not in others [43-45]. Our study also has mixed results showing a lack of effect on the primary outcome but significant effects on some of the secondary outcomes. Most of the RCT studies in the recent systematic review of practice facilitators are small compared with our study, and the outcomes are primarily assessed by questionnaires and patient record audits in contrast to data from administrative registers, which are more independent of potential interests in the intervention.

In our study, the intervention consisted of up to three visits within a year. It was an essential component in the intervention that the practices were to work with the planned topics between the visits, for example, by designing and implementing new procedures and implementing the Sentinel Data Capture. In a Danish primary care context this is considered to be an intensive intervention. Nevertheless, compared with other practice facilitation studies [20,36,37,43,45], the intervention in this study can be regarded as being of low intensity. A systematic review of practice facilitation in primary care found that the number of facilitator visits varies substantially between interventions, from a few meetings to several meetings a month throughout a whole year [11]. While some studies with few visits do report an effect [30,46,47], the review finds a positive relationship between the effect of the interventions and the intensity of visits, but not between the effect and duration of the intervention [11]. Thus, the limited effects of the present intervention may partly be ascribed to the intervention’s relatively low intensity.

In this intervention the facilitators were all general practitioners, apart from one. Hence they were colleagues and had an understanding of the organization and conditions in general practice, and they had experience of change processes from their own practices. This was considered by the project initiator as critical to ensure the participation of the practices. Compared with previous studies, it is unusual that the facilitators were GPs (although a few other studies from Denmark have used GPs as facilitators [15,33]). Most often the facilitators are practice nurses or practice assistants [11]. In several studies, however, the profession of the facilitator is not mentioned [11]. Our study was not designed to assess the influence of the profession of the facilitators.

Although the concept of facilitation is widely used, it is not well defined. Many different terms are used to describe facilitator-based interventions (e.g. “facilitators”, “educational facilitators”, “educational outreach visits”, “outreach facilitation”, “practice facilitators” and “academic detailing”) [17] and often similar terms are used to describe interventions that appear to be different. Thus, the studies included in the recent systematic review vary on several parameters such as the number of visits, the professional background of the facilitator, the flexibility of the intervention, the role of the facilitator, and the outcome measures applied. Further, the descriptions of the interventions are often superficial, which makes it difficult to assess the actual interventions and the variations between them. This seriously impedes efforts to assess the general effectiveness of practice facilitation. Thus, in practice “facilitation” can be translated (i.e. interpreted and enacted) in many ways by the participating actors during the design and implementation of a facilitator-based intervention. The translation involves both a structural-logistic aspect (related to the number of visits, the duration of the intervention and the profession of the facilitators) and a more conceptual aspect related to the facilitator’s role and the type of interaction with the practices. In addition to the lack of conceptual clarity, only few studies, such as the ones by Hogg et al., Watkins et al. and Baskerville et al., have undertaken process evaluation or studied the implementation of practice facilitation [10,18,29]. It has been recommended to undertake more process evaluations to better understand the learning processes related to facilitator visits and the variations in results from different effect studies [25]. Further, even fewer studies have explored how the role of the facilitator is carried out in practice [15,27].

In future studies it is therefore crucial that both the intended and the implemented translation of the concept are thoroughly described. Hence, we also collected a range of qualitative data alongside the RCT to explore how the concept of facilitation and the role of the facilitator were translated at all levels of the intervention from project developers to the actual visits, and how and why changes were occurring (or not occurring) during and after the visits (study not completed).

## Conclusion

The mixed results from this study indicate that a semi-tailored facilitator-based intervention of relatively low intensity is unlikely to add substantially to the implementation of disease management programmes for DM2 and COPD in a context marked by important concurrent initiatives (including financial incentives and mandatory registry participation) aimed at moving all practices towards changes in chronic care. The study also points to the importance of conducting RCTs when evaluating practice facilitation in a changing professional context because of an otherwise profound risk of overestimating the effect of a new intervention.

## Abbreviations

COPD: Chronic obstructive pulmonary disease; DAK-E: Danish Quality Unit of General Practice; DMPs: Disease management programmes; DM2: Type 2 diabetes; DNHSR: Danish National Health Service Register; GP: General practitioner; ICPC: International classification method for primary care encounters; IQR: Interquartile range; RCT: Randomised controlled trial.

## Competing interests

The authors declare that they have no competing interests.

## Authors’ contributions

TDD participated in the design of the trial, management of the trial and wrote the first manuscript draft. TT initiated the trial and participated in the design. MBK initiated the trial and participated in the design. VS participated in the design and did the statistical analysis. FBW initiated the trial and participated in the design and management of the trial. All authors contributed to drafting and revising of the manuscript. All authors approved the manuscript.

## Pre-publication history

The pre-publication history for this paper can be accessed here:

http://www.biomedcentral.com/1471-2296/15/65/prepub
